# Implementation of population screening for colorectal cancer by repeated fecal occult blood test in the Netherlands

**DOI:** 10.1186/1471-230X-9-28

**Published:** 2009-04-24

**Authors:** Maaike J Denters, Marije Deutekom, Paul Fockens, Patrick MM Bossuyt, Evelien Dekker

**Affiliations:** 1 Department of Gastroenterology, Academic Medical Centre, Amsterdam, the Netherlands; 2 Department of Social Medicine, Academic Medical Centre, Amsterdam, the Netherlands; 3 Department of Clinical Epidemiology and Biostatistics, Academic Medical Centre, Amsterdam, the Netherlands

## Abstract

**Background:**

Colorectal cancer (CRC) is the third most prevalent type of cancer in the world. Its prognosis is closely related to the disease stage at the time of diagnosis. Early detection of symptomless CRC or precursor lesions through population screening could reduce CRC mortality. However, screening programs are only effective if enough people are willing to participate. This study aims to asses the uptake of a second round of fecal occult blood test (FOBt) based screening and to explore factors that could potentially increase this uptake.

**Methods and design:**

Two years after the first screening round, 10.000 average risk persons, aged 50 to 75, will again receive an invitation to participate in immunohistochemical FOBt (iFOBt) based screening. Eligible persons will be recruited through a city population database. Invitees will be randomized to receive either an iFOBt with a faeces collection paper or an iFOBt without a collection paper. The iFOBts will be analyzed in a specialized laboratory at the Academic Medical Centre. Positive iFOBts will be followed by a consultation at our outpatient clinic and, in the absence of contra-indications and after informed consent, by a colonoscopy. The primary outcome measure is the participation rate. Secondary outcome measures are the effect of the addition of a collection paper on the participation rate, reasons for participation and non-participation, measures of informed choice and psychological consequences of screening and measures of psychological and physical burden associated with the iFOBt and the colonoscopy. Another secondary outcome measure is the diagnostic yield of the program.

**Discussion:**

In order to implement population screening for colorectal cancer in the Netherlands, information is needed on the uptake of repeated rounds of FOBt-based screening and on factors that could potentially increase this uptake in the future since effectiveness of such a program depends on the willingness of persons to participate. This study will provide information on the actual uptake and perception of a second round of iFOBt-based screening. The results of this study will contribute to the future implementation of a national colorectal screening program in the Netherlands.

**Trial registration:**

Dutch Trial register: NTR1327

## Background

Colorectal cancer (CRC) is the third most prevalent type of cancer in the world. Each year more than 940.000 persons are diagnosed with CRC and 500.000 persons die from the disease [1]. In the Netherlands, CRC accounted for over 4700 deaths in 2007[2,3]. The prognosis of patients with CRC is closely related to the disease stage at the time of the diagnosis. Five-year survival is 90% in localized disease but only 68% for disease with lymph node involvement. If distant metastases are present, survival drops to 10%[4]. Many patients are diagnosed in an advanced stage, thus resulting in poor survival. Early detection of symptomless CRC or its precursor lesions by population screening could reduce CRC mortality since removal of these precursors during colonoscopy reduces the incidence of CRC[5].

There are several options for CRC screening falling into two broad categories: stool tests and structural exams. Stool tests include fecal occult blood tests (FOBt) and tests for exfoliated DNA. Structural exams include flexible sigmoidoscopy, total colonoscopy and computed tomographic colonography (CTC). FOBt is the only screening method with a documented CRC mortality reduction in randomized controlled trials. The efficacy of strategies based on repeated guaiac FOBt has been established in three randomized trials and one non-randomized controlled trial [6-9]. A meta-analysis after several rounds of these continuing trials showed a CRC mortality reduction of 14% during a 10-year screening period[10].

The United Kingdom, France, Finland and Australia provide nationally organized bowel cancer screening programs. Several other countries, including Germany, the United States and Italy, recommend CRC screening but do not offer screening as part of an organized national program. In the past few years, several project-groups have started to study the feasibility, acceptance and cost-effectiveness of a national colorectal cancer screening program in the Netherlands. In 2006 our research group, in collaboration with the Radboud Hospital in Nijmegen, started a study on the implementation of invitation based population screening with FOBt in two regions of the Netherlands. In this pilot study, 20.000 asymptomatic individuals, aged 50–75, were invited to take part in one round of FOB testing and they were randomized to either a guaiac-based (gFOBt) or immunochemical FOBt (iFOBt). Invitation of participants was from May 2006 until January 2007. Primary goals of this implementation study were to determine the participation rate, feasibility and logistics of this type of screening. In this study, an overall participation rate of 53% was observed. Participation rate in the iFOBt group was significantly higher than in the gFOBt group (60% vs 47%)[11]. Also, detection rates for advanced adenomas and cancer were higher for iFOBt although also more colonoscopies had to be performed.

Since the effectiveness of a FOBt-based screening program in reducing mortality from CRC is highly dependent on participants' willingness to repeat testing at regular intervals, there is a need for information on adherence to consecutive rounds of FOBt screening. Information on actual uptake is needed before well-informed decisions on nationwide introduction of a CRC screening programme in the Netherlands can be made.

To obtain information on participants' willingness to take part in repeated rounds of FOBt-based screening we decided to add a second screening round to the pilot study described above. In the present study the same cohort of the Amsterdam region that was studied in the first screening round will be again invited for FOBt-based screening after an interval of two years. Only the iFOBt will be used in this second round since it was associated with a higher participation rate in our first round and it had higher CRC detection rates. By focusing on the exact same group of persons we intend to represent the real life situation once a nationwide program is in place.

Participation rate will be the major focus of the present study but we will also try to identify factors that are associated with participation and non-participation. Particularly, we want to explore whether the addition of a collection paper makes people more willing to participate. It is hypothesized that the handling of faeces is a barrier to participation. Facilitating the collection process by adding a collection paper could potentially lower this barrier and increase participation.

Besides pursuing a high participation rate, it is also important that invitees are enabled to make a well-informed decision on participation. In order to make an informed decision invitees need to have sufficient insight into the pros and cons of taking part in the program. This study will therefore also evaluate to what extent invitees' decisions to participate or not are made in a well-informed fashion. Furthermore, the psychological and physical impact of taking part in the screening program and possible follow-up investigations will be evaluated. Insight into the impact of participation is important because negative experiences can act as a barrier for future participation. Finally, the diagnostic yield of this second round of screening will be evaluated and compared to the first round. It is hypothesized that the majority of advanced lesions were already detected during the first round. Therefore we expect to find a smaller number of patients with advanced lesions compared to the first round.

## Methods and design

### Objectives

#### Primary objective

To evaluate the participation rate in a second round of iFOBt-based colorectal cancer screening in the Netherlands, two years after the first round.

#### Secondary objectives

• To evaluate the effect of the addition of a faeces collection paper to the iFOBt-kit on the participation rate;

• To compare participation rates between the first and second round;

• To compare the participation rates in groups that were defined as participants or non-participants in the first round;

• To evaluate the diagnostic yield (detection rates of cancer, high-risk adenoma and low-risk adenoma) and complication rate of iFOBt and colonoscopy and to compare these to results of the first screening round;

• To compare the baseline characteristics of participants and non-participants;

• To evaluate factors influencing the decision to participate;

• To evaluate invitees' ability to make a well-informed decision to participate;

• To evaluate participants' experience with the screening program in terms of their perception and the burden of the iFOBt;

• To evaluate the psychological impact on participants of receiving a positive test result;

• To evaluate the burden of the colonoscopy in iFOBt positives;

### Study design and randomization

The study design is a cohort study examining the participation rate of a second round of FOBt screening. Within the cohort study a randomized controlled trial will be executed comparing the use of a collection paper versus no collection paper on the participation rate. All persons eligible for invitation are pre-randomized to one of both groups before informed-consent is obtained. Through randomization, non-responders to the previous round, responders to the previous round and first-time invitees will be equally distributed over both study groups.

### Study population

Our study population consists of 10.000 average risk persons between 50 and 75 years of age with the postal code of our target area. The target area of the second round is identical to the target area of the first round and is selected because it had an average uptake in the nationwide breast cancer screening program. Thus, among the invitees of the second round 3 different groups can be identified: 1) non-responders to the first round; 2) responders to the first round; 3) first-time invitees (persons aged 50–75 that moved into the target area or persons that turned 50 within the last 2 years).

Institutionalized people will be excluded from participation. Responders to the first round that tested positive in the first round and had a follow-up colonoscopy will not be re-invited. They will be advised follow-up conform the national guidelines on surveillance colonoscopy[12].

Other exclusion criteria are CRC symptoms in the last three months (rectal blood loss and/or changed bowel habits and/or unintentional weight loss). These symptomatic persons will be advised to contact their general physician and not to participate in the study. Also, persons with a life-expectancy of less than 5 years and persons that have undergone a colonoscopy in the previous 2 years will be excluded from participation.

### Invitation procedure

Invitation will take place from August 2008 until June 2009. The screening program uses a centralized invitation procedure and all invitations will be sent out by the regional Comprehensive Cancer Centre Amsterdam. This institution is also involved in the organization of other population-based cancer screening programmes in the region including breast and cervical cancer screening. All persons will receive an invitation kit by postal mail containing an invitation letter, an information leaflet, an immunochemical FOBt, a test instruction and a frequently asked questions card. A collection paper to facilitate the collection of faeces will be enclosed in half of the invitation kits.

A reminder will be sent after two and eight weeks.

### Information leaflet

The information leaflet is an updated version of the leaflet that was used in the first round. The main difference is that it is adjusted following the principle of informed choice. This principle states that in order to enable a person to make a well-informed decision to participate in a screening program or not, certain information has to be available to this person. The information that was already present in the previous leaflet is optimized and covers all information relevant to colorectal screening: details of CRC, details of the iFOBt, possibility of false-negative and false-positive results, details of the follow-up investigation colonoscopy, prognosis and treatment options, and details of benefits and harms. Also, the freedom of choice to participate in the program or not is emphasized.

### iFOBt

The iFOBt that will be used in this study is the OC-sensor (Eiken Chemical Co, Tokyo, Japan). This is the same iFOBt that was used in the first round. This is a clinical laboratory-based, automated, immunochemical test that measures human haemoglobin content of a stool sample. No diet restrictions are necessary before performing the test.

The iFOBt consists of a sampling bottle with a sampling probe attached to the cap. Participants are instructed to collect the faeces sample by scraping the probe over a broad area of the stool surface. After the sample is taken, the probe is reinserted into the sampling bottle. By reinserting the probe, the fecal sample is suspended in haemoglobin-stabilizing buffer. Participants are instructed to keep the bottle in a dark and cool place and to return it as soon as possible.

Returned iFOBts will be collected at the specialized laboratory of the AMC and will be processed by quantitative haemoglobin analysis by the OC-MICRO automated instrument using a 50 ng Hb/mL threshold to determine positivity.

### Collection paper

All persons randomized to the collection paper group will receive a floating collection paper (Eiken Chemical Co, Tokyo, Japan) with their iFOBt. This is a biodegradable disposable paper float that has to be placed in the toilet bowl to immobilize the stool for easy sampling.

### Informed consent

Written informed consent will be obtained from all participants before analyzing the stool sample. Enclosed in the invitation kit is an informed consent form that participants are asked to return together with the test bottle. Test bottles will not be processed in the absence of a signed informed consent. When the informed consent is lacking, a notification will be sent to the participant together with a request to return the informed consent form.

### Provision of test results

All specimens will be analyzed in a specialized laboratory. Participants will receive the test result by postal mail. A positive test result is defined as an Hb content of 50 ng/mL or more. In this case the participant will be invited for a consultation at the colorectal screening centre in the AMC to discuss the recommended follow-up investigation: the colonoscopy. In case of a negative test result no follow-up is needed. In case of a positive test result, the general practitioner will also be informed.

### Follow-up colonoscopy

During the consultation at the colorectal screening centre, information is given on the consequences of the positive test result. In absence of any contraindications a colonoscopy will be advised and all relevant information on this examination (technique, risks and alternatives) will be given. If the participant consents, a colonoscopy will be performed within two weeks after the consultation.

Colonoscopy is considered the clinical reference standard for detection of adenomatous polyps and CRC. The procedure will be performed in our screening centre according to quality guidelines, adapted from the recently published guidelines of the American Society for Gastrointestinal Endoscopy[13]. In case of polyps or cancer, endoscopic removal of the lesion will be attempted during the same procedure. If immediate endoscopic treatment is impossible, biopsies will be obtained and pathological assessment of these tissue samples will provide definitive diagnosis so further treatment can be initiated.

### Lesions

Of all lesions, data on location (centimetres from anus, segment of the colon), size (millimetres) macroscopic aspect (hyperplastic, adenomatous, carcinomatous), morphology (sessile, pedunculated, flat, depressed), diagnostic or therapeutic procedure (total polypectomy, piecemeal polypectomy, biopsy), use of saline and/or epinephrine), and macroscopic involvement of margins will be recorded during the colonoscopy.

### Pathology

One experienced gastrointestinal pathologist will evaluate all samples. Of each lesion the histology (hyperplastic polyp, serrated adenoma, tubular adenoma, tubulovillous adenoma, villous adenoma or carcinoma,) and grade of dysplasia (low- or high-grade) will be assessed according to the Vienna classification.

### Follow-up after colonoscopy

The findings at colonoscopy will be discussed two weeks after the colonoscopy through a consultation by telephone or at the outpatient clinic. A confirmation of the colonoscopy result will be sent by postal mail. In case of a negative colonoscopy, no follow-up is needed. Follow-up of patients after removal of adenomatous polyps or cancer will be performed by surveillance colonoscopies according to the Dutch CBO consensus[12]. Cases of CRC will be referred for appropriate management (e.g. consultation of a surgeon). The general practitioner will also receive a report of the colonoscopy procedure.

### Questionnaires

#### Socioeconomic and demographic characteristics

Baseline characteristics as age, marital status, education, employment and ethnicity will be recorded by a questionnaire two weeks after the invitation is sent.

#### Informed choice

Informed choice (knowledge and attitude) will be measured at baseline.

To evaluate whether the principle of informed choice applies, it is necessary to understand the knowledge, attitude and behaviour of the invited persons. The decision to participate in screening will be classified as an informed choice if: a) the participant has a positive attitude towards undergoing a test; b) the participant has relevant knowledge about the test. Knowledge is considered relevant if it covers 12 general domains. (These domains are applicable to all types of mass screening and are adapted from the Dutch prenatal screening for Down syndrome[14]) and c) this person actually undergoes the test. The decision not to participate in screening will be classified as an informed choice if: a) an individual has a negative attitude towards undergoing a test; b) an individual has relevant knowledge about the test; and c) this person does not undergo the test. All choices that occur when individuals do not have relevant knowledge or when their attitudes are not reflected in their behaviour are to be considered uninformed[15].

Knowledge about screening with iFOBt will be measured using 24 statements derived from expert opinion and information obtained in the first screening round, with two response options (e.g. "the fecal occult blood test has to be repeated every 2 years" [correct-incorrect]). The number of correct items is used as the knowledge score. Attitude regarding undergoing screening will be measured by 4 items, each scored on a 7-point scale (e.g. "for me, having the screening test for colorectal cancer would be...." [a good idea-a bad idea]. Attitude items are based on the Marteau's measure of informed choice[15]. Screening behaviour is measured by either participation or non-participation in the present study (i.e. returning of the iFOBt).

#### Reasons for (non)participation

Reasons for (non)participation will be collected in all invitees at baseline.

For the present study the Health Belief Model (HBM)[16] will be used as theoretical background to understand reasons for (non-)participation. This model is widely used to explain cancer screening adherence. The HBM states that health behaviour depends on an individual's desire to avoid illness and the belief that a specific action will prevent illness. Key factors in explaining health behaviour are: perceived susceptibility, perceived severity, self-efficacy, perceived benefits, perceived barriers. Reasons for (non)participation will be measured by eight items scored on a 4-point scale (e.g. "I think I have a higher chance of having colorectal cancer than other people my age..." [totally agree-totally disagree]. These questions are supplemented with 12 questions used in the first screening round (e.g. "I find that performing the FOBt is disgusting..." [agree – not agree].

#### Psychological consequences of a positive FOBt

Psychological consequences of screening will be documented in all invitees at baseline and immediately before and 2 weeks after undergoing colonoscopy in participants with a positive FOB test result.

The psychological consequences of receiving a positive FOB test result will be measured with the validated Dutch version of the 'Psychological Consequences of screening Questionnaire' (PCQ)[17]. The English version of this questionnaire has successfully been used in various screening studies[18]. The PCQ consists of three subscales: emotion (5 items), physical dysfunction (4 items) and social dysfunction (3 items). Scores on all items vary between 0 (not at all) and 3 (often) (e.g. "during the past week I had problems sleeping..." [not at all – often].

#### Burden of colonoscopy

The burden of undergoing a colonoscopy will be measured two weeks after the colonoscopy. Participants will be asked to rate on a 5-point scale the degree of embarrassment, pain and burden for one of several aspects of the colonoscopy procedure. Satisfaction with the procedure will be measured by 20 statements scored on a 4-point scale (e.g. "the endoscopist treated me with respect..." [not agree – agree ]. These 20 items-of-concern were developed from focus group sessions with patients regularly undergoing colonoscopies.

### Ethical approval

Ethical approval was obtained by the Committee on the Population Screening Act (WBO Committee).

### Data analysis

The primary goal of this second FOBt-based screening round is to assess the overall participation rate and to compare participation rates between groups (participants (1) and non-participants (2) to the first round and new invitees (3), within all three groups invitees will be randomized to receive a collection paper or not).

Group differences will be calculated using the Chi-square test. Descriptive statistics will be used to compare baseline characteristics of the subgroups.

To assess the effect of baseline characteristics and independent variables (assessed in the questionnaires) on the participation rate, multivariate analysis will be performed.

### Sample size

In the first round 10.000 persons were invited for screening. Since we are focussing on the exact same group of persons, the number of persons that will be invited for the current study will also be 10.000.

Anticipating a 50% participation rate and the invitation of 10.000 eligible persons in the current study, the participation rate and its 95% confidence interval can be estimated. Assuming a participation rate of around 50%, the two-sided 95% confidence interval for the participation rate will extend 1% from the observed proportion, using the large sample approximation to the binomial distribution.

This sample size will allow us to detect differences of approximately 3% in participation rate between subgroups, with a continuity corrected chi-square test and a 0.05 two-sided significance level, requesting a power of 80%.

## Discussion

This study will provide information on the uptake of a colorectal cancer screening program using repeated immunochemical FOBt in the Netherlands. These data are crucial since reliable and precise data on the degree to which people are willing to participate in a second round of screening in the Netherlands are lacking and the effectiveness of a screening program is directly influenced by the participation rate. Insight into factors that are related to participation in biannual screening can be used to optimize future screening programs. If this study shows that a less burdensome faeces collection procedure by the use of a collection paper has a positive effect on the participation rate, this information is useful for a future population screening program. Furthermore, we can anticipate on the psychological and physical burden of participating in a screening program based on participant's experiences in the present study. In a future screening program this burden should be reduced to a minimum to ensure a high participation rate.

## Abbreviations

CRC: colorectal cancer; AMC: Academic Medical Centre (Amsterdam); FOBt: fecal occult blood test; iFOBt: immunochemical fecal occult blood test; gFOBt: guiac fecal occult blood test; PCQ: psychological consequences of screening questionnaire.

## Competing interests

The authors declare that they have no competing interests.

## Authors' contributions

MD is responsible for the drafting of the manuscript. ED, MarD, PF and PB are responsible for the study design and the revision of the manuscript. All authors have read and approved the manuscript.

## Pre-publication history

The pre-publication history for this paper can be accessed here:

http://www.biomedcentral.com/1471-230X/9/28/prepub
